# Supplementary material for: Injectable and biodegradable piezoelectric hydrogel for osteoarthritis treatment
Source: Nat Commun. 2023 Oct 6;14:6257. doi: 10.1038/s41467-023-41594-y (PMC10558537; doi:10.1038/s41467-023-41594-y)
Supplement: Supplementary file 1 — Supplementary Information [file 41467_2023_41594_MOESM1_ESM.pdf]

Supplementary Information for

Injectable and Biodegradable Piezoelectric Hydrogel for Osteoarthritis Treatment

Tra Vinikoor, Godwin K. Dzidotor, Thinh T. Le, Yang Liu, Ho-Man Kan, Srimanta Barui, Meysam T. Chorsi, Eli J. Curry, Emily Reinhardt, Hanzhang Wang, Parbeen Singh, Marc A. Merriman, Ethan D'Orio, Jinyoung Park, Shuyang Xiao, James H. Chapman, Feng Lin, Cao-Sang Truong, Somasundaram Prasadh, Lisa Chuba, Shaelyn Killoh, Seok-Woo Lee, Qian Wu, Ramaswamy M. Chidambaram, Kevin W. H. Lo, Cato T. Laurencin, Thanh D. Nguyen*

*Corresponding author: nguyentd@uconn.edu (Thanh D. Nguyen)

<u>Contents</u>:

Supplementary discussions

Supplementary Figure 1: Schematic illustration of piezoelectric nanofibers fabrication process.
Supplementary Figure 2: Characterization of the short nanofibers of PLLA (NF-sPLLA) and piezoelectric hydrogel.
Supplementary Figure 3: Piezoelectric hydrogel *in vitro* biocompatibility assessment.
Supplementary Figure 4: *In vitro* assessment on chondrogenesis, induced by piezoelectric hydrogel and US activation.
Supplementary Figure 5: Output voltage (Vpp) of dried NF-sPLLA hydrogel sensors at various concentrations.
Supplementary Figure 6: Piezoelectric hydrogel reduces *TNF alpha* expression.
Supplementary Figure 7: Piezoelectric hydrogel for OC treatment on rabbit study.
Supplementary Figure 8: Piezoelectric hydrogel for cartilage healing evaluated by chondrocyte hypertrophy.
Supplementary Figure 9: Piezoelectric hydrogel induces cartilage healing evaluated by cell apoptosis using H&E.

Supplementary Tables 1: ICRS macroscopic evaluation of cartilage repair.
Supplementary Tables 2: ICRS visual histological assessment scale.

Supplementary References.

Supplementary Discussion: Piezoelectricity stimulation using ultrasound activation *in vivo*.

Penetration assessment of ultrasound at the defect site.

To validate whether 40 kHz US, 0.33 Watt/cm^2 can penetrate through various tissues, including skin, muscle, and ligament, and reach the targeted defect site, we performed US penetration experiments on cadaver rabbit knee. We selected lead zirconate titanate (PZT) as a positive control because PZT is a commonly used piezoelectric material for many medical applications. When using a 40 KHz US transducer to measure piezoelectric response of materials, the carried-out data may entangle with electromagnetic interference (EMI) noise. Therefore, we utilized non-piezoelectric material (e.g., polyimide) to generate a baseline which is only subjective to EMI but does not exhibit piezoelectric properties. As seen in Supplementary Figure 7.b the polyimide sensor also shows some signals at 40 kHz, but purely EMI noise and not piezoelectric signal. However, PLLA sensor which has comparable dielectric constant to polyimide (2.7 for PLLA and ~3 for polyimide), produced significantly higher output voltage due to its piezoelectricity properties. Supplementary Figure 7.b demonstrates that the 40 kHz US can effectively penetrate different tissues and reach the intended defect site, successfully activating the piezoelectric response of the PZT and PLLA sensors.

PZT possesses greater piezoelectric constants than PLLA which often leads to an assumption that PZT produces a much higher output than PLLA. However, this phenomenon is only correct when the materials are stimulated by impact forces at low frequencies. For ultrasound transmission, especially for responding to the US, the output performance of the piezoelectric materials depends strongly on their acoustic impedance, which is defined as how well the US can be transmitted between different mediums (in our case, between tissues and the testing sensor). Indeed, PZT acoustic impedance is very high (34.7 MRayl) compared to averaged acoustic impedance of tissues (~1.5 MRayl), leading to a major of US scattered or reflected to surrounding tissues instead of stimulating the material. In fact, for practical applications, PZT-based ultrasound transducers require a matching layer and a backing layer to receive/respond to the US effectively. On the other hand, PLLA's acoustic impedance is (~2.3 MRayl), which is closer to one of the tissues, allowing more US can activate the materials. Therefore, the signal from the PLLA sensors is only slightly smaller than PZT sensors in our ultrasound measurement.

Ultrasound parameters selection to activate piezoelectric hydrogel in knee joint.

In vivo, US frequency and intensity experiments were maintained identical to those utilized for *in vitro* studies, consisting of a 40 KHz ultrasound (US), 0.33 Watt/cm^2 and exposure for a duration of 20 minutes. We kept using these settings because 1) although 1-3 MHz US frequencies are commonly utilized for US therapy, to penetrate through knee joint and activate the piezoelectric properties of NF-sPLLA hydrogel, a low frequency (e.g., 40 kHz) is more suitable. This is because a lower tissue absorption rate is observed at lower frequencies. Regardless of the frequency employed, it is crucial to ensure that the intensity remains below 0.5 Watt/cm^2, as low-intensity US is considered safe for human use [1-4]. 2) The *in vitro* data (Figure 2. a-f) clearly demonstrates that the chosen US parameters were efficient in activating electrical charge in the Piezo hydrogel. This efficiency is evidenced by the upregulation of gene expressions (*COL2A*, *ACAN*, and *SOX9*), as well as the increased formation of GAG and Collagen II protein in the *Piezo + US* group, when compared with the control/sham groups. 3) We also verified that the same US parameters applied in our study effectively activated the piezoelectric charge within the knee joints, as illustrated in

Supplementary Figure 7.b. This additional evidence further supports the rationale behind our chosen US parameters for *in vivo* experiments.

Safety considerations and treating dosages of ES/ piezoelectricity.

Electrical stimulation (ES) could adversely affect tissue in several ways, such as electrical burns, irreversible electroporation, and electric shock[5]. Consequently, caution must be exercised when utilizing ES for tissue engineering, as excessive ES can harm the body. The configurations of ES involve various factors, including field strength, stimulation duration, and the type of ES, such as direct current (DC) (directly contacted DC and capacitive coupling) or biphasic current (pulses and alternating current). Therefore, defining a safe threshold depends on the specific configuration of ES applied. However, there is currently a lack of clear guidelines or comprehensive studies identifying/evaluating safe threshold parameters for ES use in tissue regeneration, particularly in cartilage healing.

To establish safe parameters for piezoelectric stimulation, careful consideration of various factors is required. Firstly, for piezoelectricity activation methods, vibration intensity or mechanical pressure applied to piezoelectric material should fall within a range that mitigates the risk of cartilage damage. Secondly, the magnitude of voltage output generated by the piezoelectric stimulation must adhere to the safe range for ES. In this regard, for US intensity, we utilized low intensity (0.33 $watt.cm^{-2}$) which is safe for human use[1-4]. Furthermore, with the intensity of US employed in our study, the voltage output generated is very small and comparable to that observed in Barker's study, which utilized ES (ranging from 15 to 500 mV) for cartilage regeneration in a rabbit model[6]. More importantly, our *in vitro* data indicates that the US intensity and the resulting output voltage applied to cells are biocompatible (Supplementary Figure 3.c) and do not cause any harm to rabbits after a two-month treatment period (Supplementary Movie 4). Collectively, we believe the parameter for piezoelectric stimulation applied in this study is safe.

For chondrogenesis, electrical stimulation parameters and piezoelectricity dose vary across different studies[6,7]. Currently, there is no clear value on an optimal or effective electrical cue dose for promoting cartilage healing. However, based on our *in vitro* study, we have found that our chosen US parameters and the charge generated from our piezoelectric hydrogel are safe to promote stem cell proliferation and effective to facilitate their differentiation into chondrocyte cells. *In vivo* data including Figure 4, Figure 5 and Supplementary Figure 7 demonstrate that the same piezoelectricity dose healed critical size osteochondral defects on rabbits by increasing subchondral bone formation and regenerating organized hyaline cartilage structures that integrate well with surrounding native tissue.

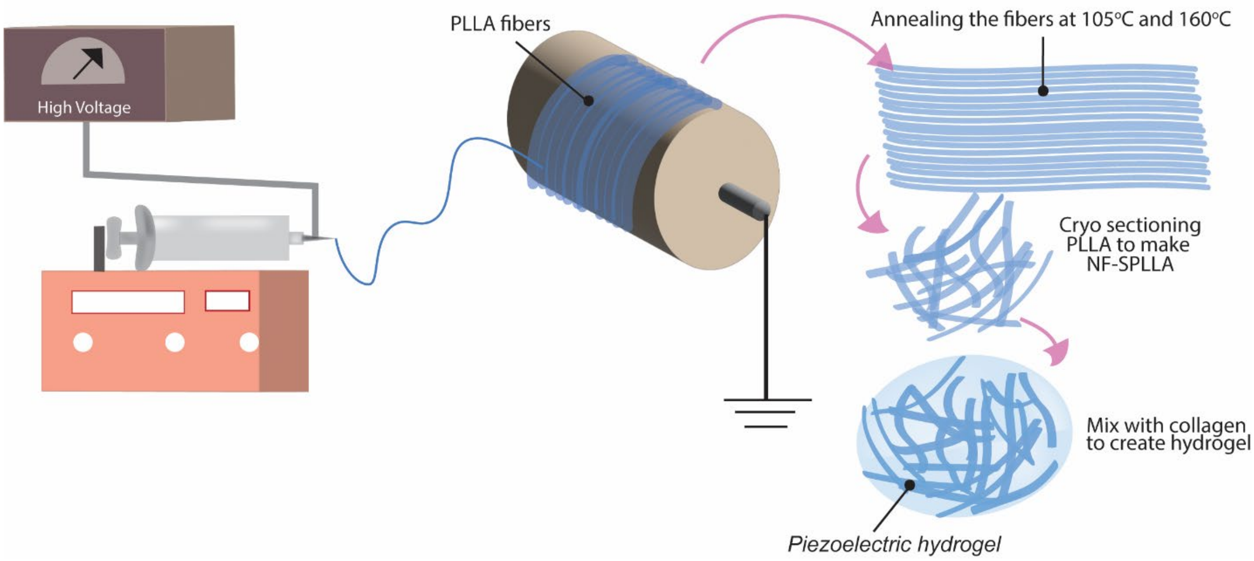

Supplementary Figure 1 | Schematic illustration of piezoelectric nanofibers fabrication process. First, PLLA fiber mat was made using electrospinning, collected on a rotating drum. The fibers mat was then annealed at 105°C and 160°C overnight, and slowly cooled down. Next, the fibers mat was embedded inside OCT gel and cryo-sectioned into short fibers (25 μm). The samples were cleaned with distilled water until clean and then lyophilized to remove the water. The collected fibers were then mixed well with collagen hydrogel.

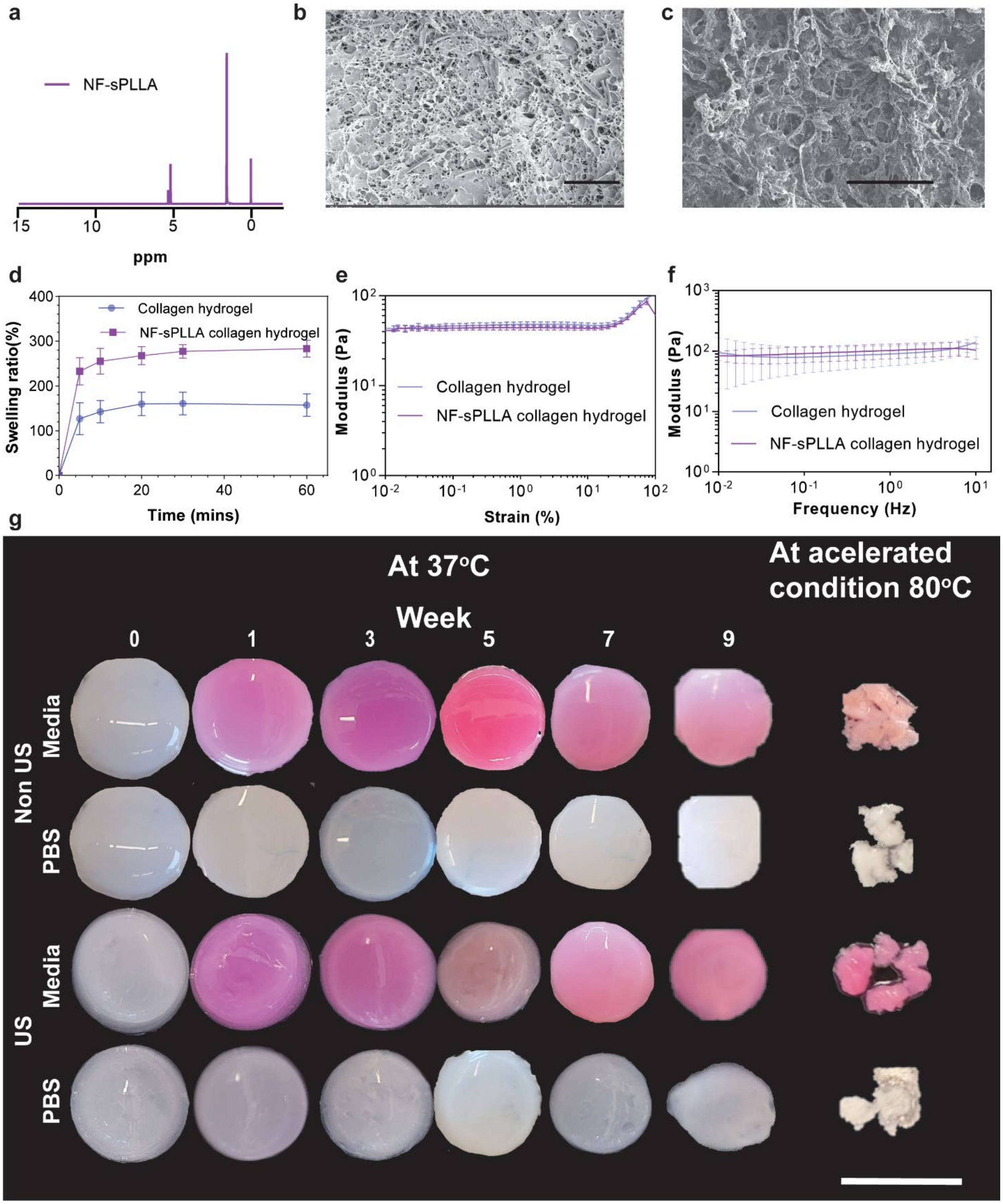

Supplementary Figure 2 | Characterization of the short nanofibers of PLLA (NF-sPLLA) and piezoelectric hydrogel. a, NMR spectra of NF-sPLLA after processing, the samples were dissolved in deuterated DCM (CD2Cl2). There was no peak for chloroform (7.26 ppm) (which was used as the solvent to dissolve PLLA for electrospinning) presented in the spectra, NF-sPLLA pattern is similar to virgin PLLA pellet in our previous study[8]. **b**, and **c,** SEM images of NF-sPLLA in collagen hydrogel and hydrogel only in dry scaffold. Incorporating PLLA fibers inside the

collagen hydrogel created more porous structure, compared to the original collagen gel (Scale bars: 50 μm). **d**, Swelling ratio profile of collagen hydrogel and PLLA short fibers in 60 mins (n=3 independent samples, the data are expressed as Mean ± SEM value). **e**, Strain sweep of collagen hydrogel and the NF-sPLLA hydrogel (n=3 independent samples, the data are expressed as Mean ± SD value). **f**, Frequency sweep of collagen hydrogel and NF-sPLLA hydrogel (n=3 independent samples, the data are expressed as Mean ± SD value). **g**, A representative photograph of 3 independent samples showing degradation ability of NF-sPLLA hydrogel at 37°C and accelerated condition 80°C both in media and PBS with and without US treatment (Scale bar: 1 cm).

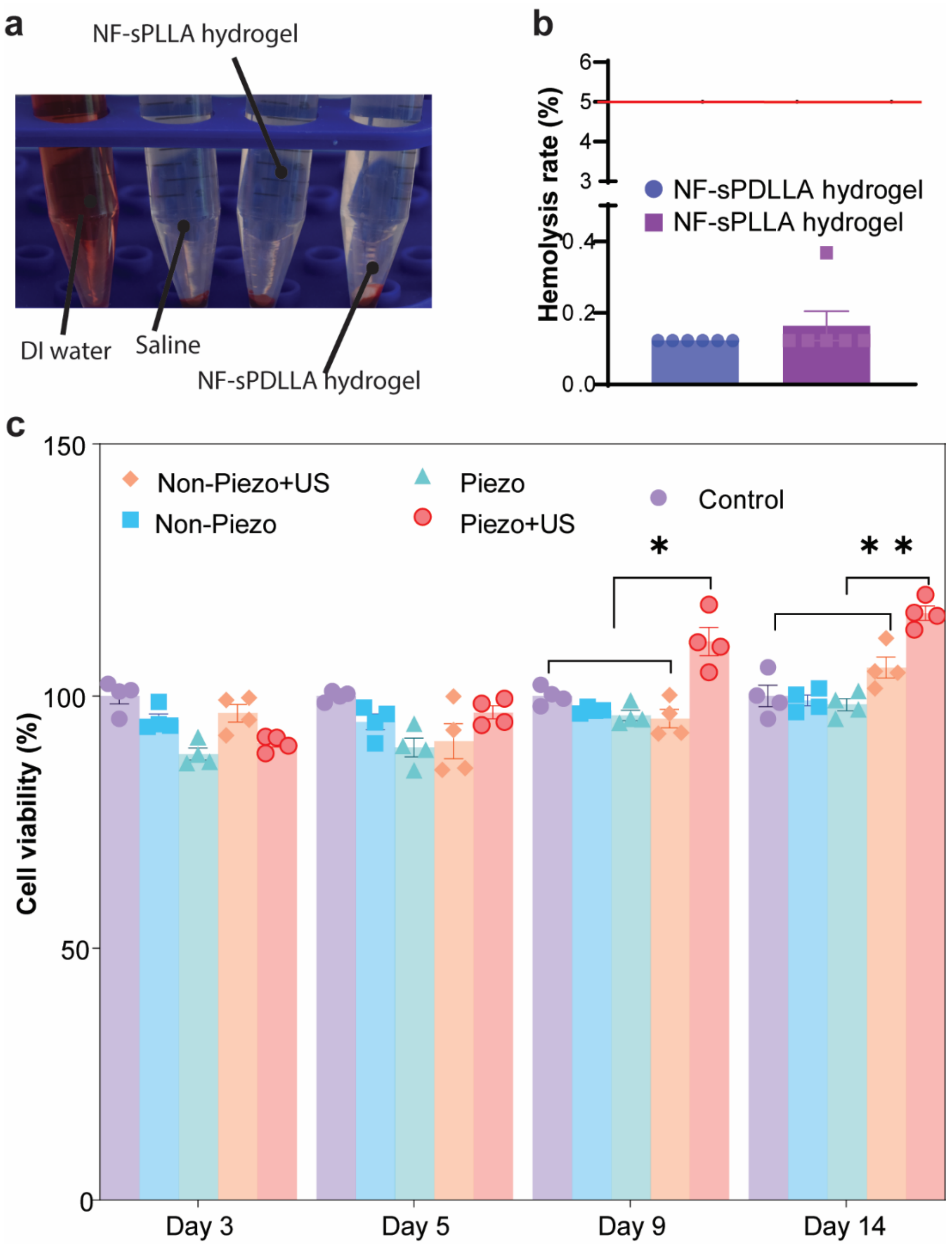

Supplementary Figure 3 | Piezoelectric hydrogel *in vitro* biocompatibility assessment. a, Photograph of hemolysis testing of NF-sPLLA collagen hydrogel, and NF-sPDLLA collagen hydrogel with normal saline as negative control and deionized (DI) water as positive control (n=6 independent samples were tested). **b,** Hemolysis rate of NF-sPLLA and NF-sPDLLA hydrogel. The red line indicates limit for implant required by International Standards Organization (ISO, standard number 10993-4) which is less than 5% (n=6 independent samples, the data are expressed as Mean ± SEM value). **c,** Viability of ADSCs seeded inside the Piezo, Non-piezo and control (collagen only) hydrogel with and without US activation for 3, 5, 9 and 14 days (n=4 independent samples, the data are expressed as Mean ± SEM value, *p<0.05, **p<0.01, one-way ANOVA, Dunnett's multiple comparisons test). Exact p-value were provided in the Source Data file.

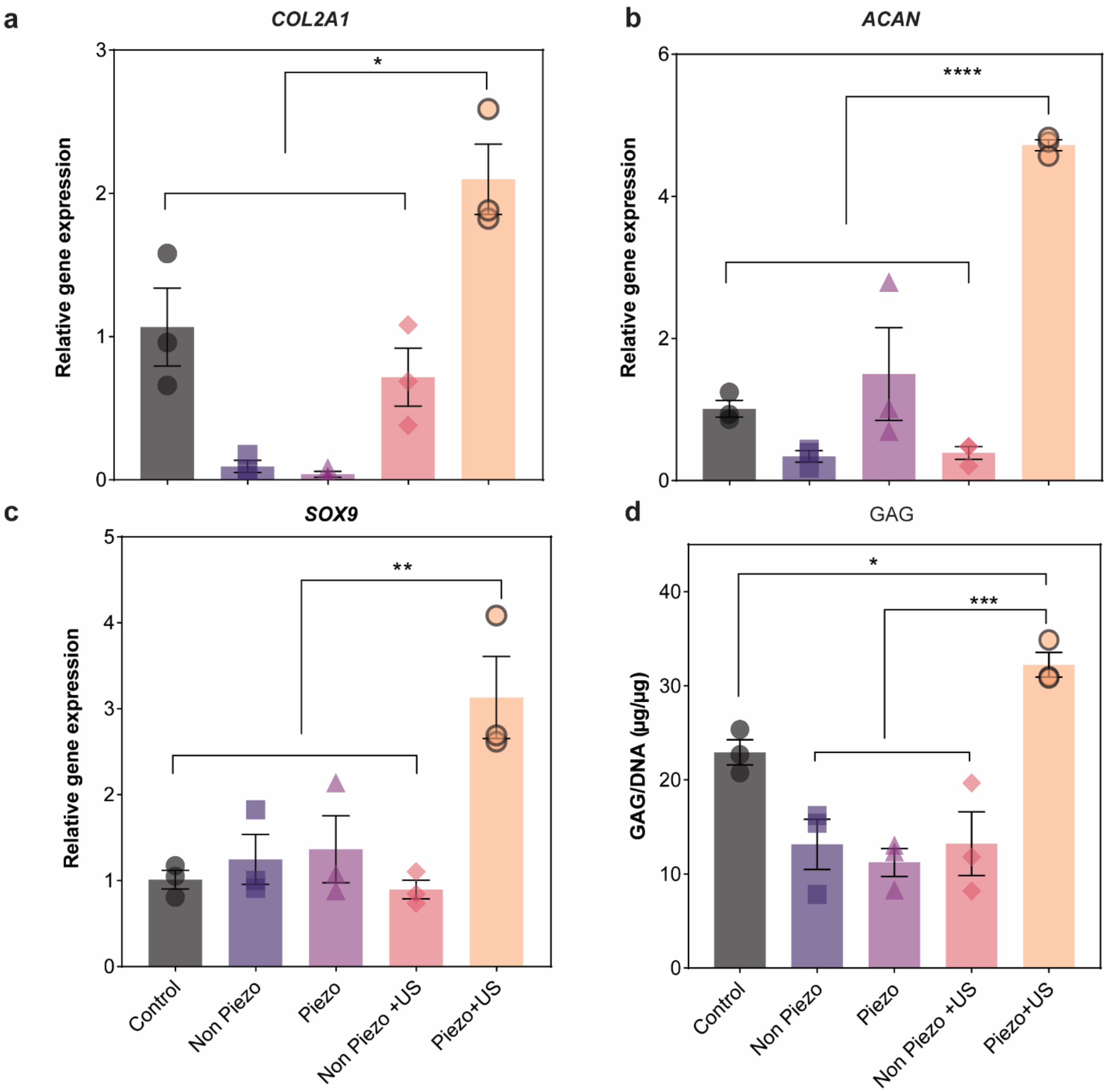

Supplementary Figure 4 | *In vitro* assessment on chondrogenesis, induced by piezoelectric hydrogel and US activation. a-c, Relative gene expression of the chondrogenesis gene markers *COL2A1*, *ACAN*, and *SOX9* in chondrogenesis medium containing DMEM supplemented with100μg/ml sodium pyruvate, 0.2 mM ascorbic acid 2-phosphate, 1% penicillin and streptomycin, 50μg/ml Insulin-Transferrin-Selenite premix (ITS). Also, before each use, dexamethasone and TGF-β3 were freshly added (n=3 independent samples, the data are expressed as Mean ± SEM value, *p < 0.05, **p < 0.01 and ****p < 0.0001, one-way ANOVA, Dunnett's multiple comparisons test). **d,** GAG/DNA (μg/ μg) ratio carried out by dimethylmethylene blue (DMMB) kit and dsDNA qualification kit, (n=3 independent samples, the data are expressed as Mean ± SEM value, *p < 0.05 and ***p<0.001, one-way ANOVA, Dunnett's multiple comparisons test). Exact p-value were provided in the Source Data file.

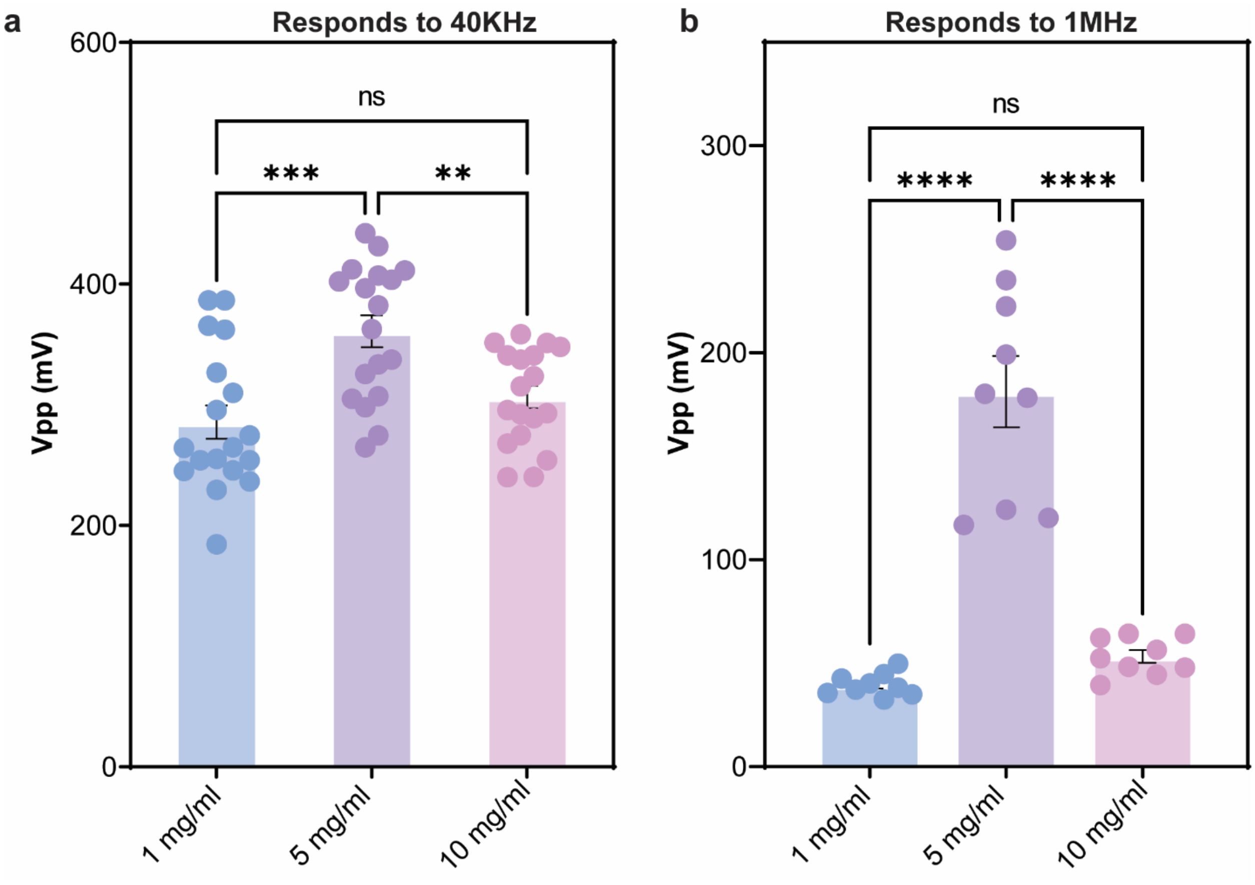

Supplementary Figure 5 | Output voltage Vpp of dried NF-sPLLA hydrogel sensors at various concentrations subject to a, 40 KHz (n=3 independent sensors, each sensor was measured one time with 9 data points were collected, the data are expressed as Mean ± SEM value, $^{**}p < 0.01$, $^{***}p < 0.001$, n.s = not significant, one-way ANOVA, Tukey's multiple comparison tests). and **b,** 1 MHz (n=3 independent sensors, each sensor measure one time with 6 data points were collected, the data are expressed as Mean ± SEM value $^{****}p < 0.0001$, n.s = not significant, one-way ANOVA, Tukey's multiple comparison tests). Exact p-value were provided in the Source Data file.

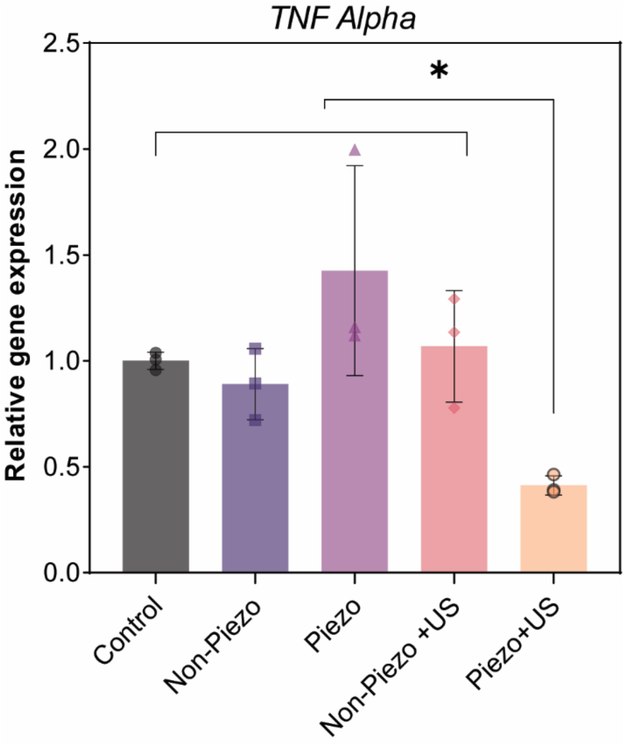

Supplementary Figure 6 | Piezoelectric hydrogel reduces *TNF alpha* expression. *TNF alpha* gene expression when co-culturing THP-1 (human monocyte) cells with ADSCs inside the Piezo, Non-piezo and control hydrogels (collagen only) with and without US activation, suggested that co-culturing ADSCs and THP-1 in Piezo hydrogel activated by US can reduce the *TNF alpha* mRNA expression, therefore reducing inflammation (n=3 independent samples, the data are expressed as boxes and points with Mean ± SEM value, $^*p < 0.05$, one-way ANOVA, Tukey's multiple comparisons test). Exact p-value were provided in the Source Data file.

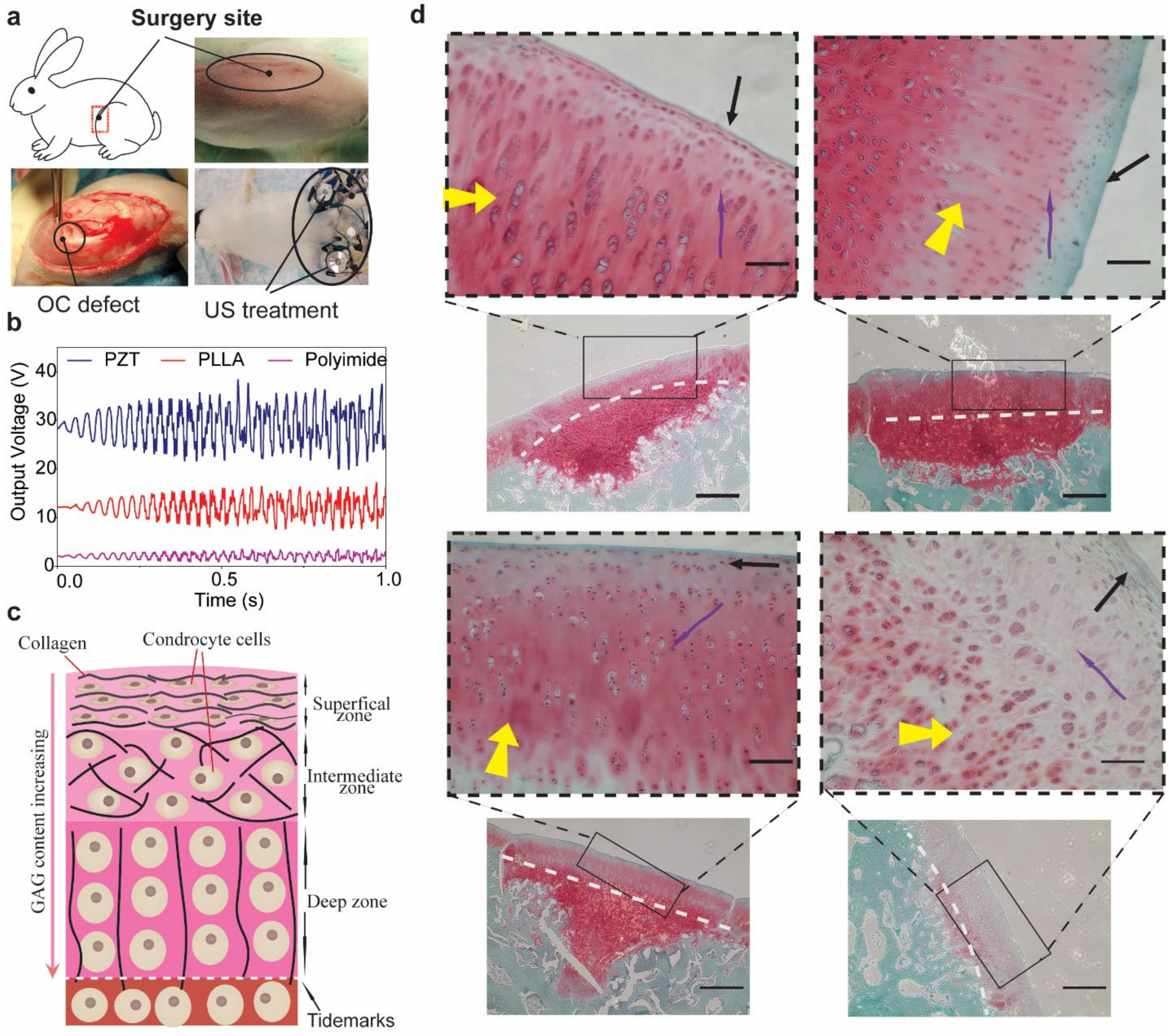

Supplementary Figure 7 | Piezoelectric hydrogel for OC treatment on rabbit study. **a**, Photographs of surgery site, critical size defect creation, and US activation treatment on rabbit knee. **b**, Results from output voltage waveforms of PZT (positive piezoelectric group), piezoelectric PLLA (experimental group), and polyimide (negative control) sensors inside the rabbit joint and activated by the US. **c,** Diagram of hyaline cartilage structure. **d**, Safranin O/fast green staining showing hyaline cartilage structure of experimental group (n=4 knee joints) after 2 months of treatment. Black arrows indicate the superficial zone with a high number of flattened chondrocyte cells, violet arrows indicate intermediate zone with spherical chondrocytes, yellow arrows indicate deep zone with chondrocytes orientated in column-like stacks and white dash lines indicate tidemarks (Scale bars: 200 µm, for zoom in image, scale bars: 100 µm).

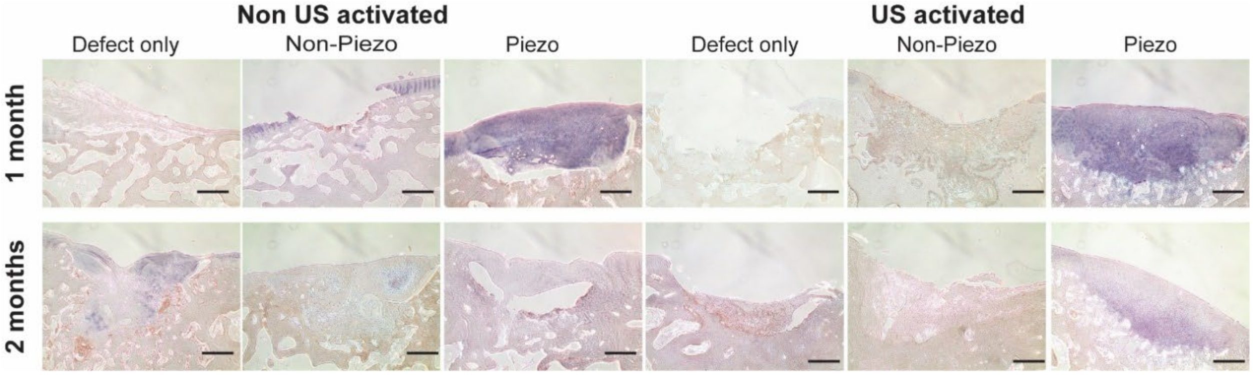

Supplementary Figure 8 | Piezoelectric hydrogel for cartilage healing evaluated by chondrocyte hypertrophy. Using collagen X staining to evaluate the hypertrophic chondrocyte for sham (defect only), non-piezo/piezo hydrogels with and without US activation (1-2 months). Collagen X was identified as dark brown color and Non-collagen X, background was identified as lavender color. In the Piezo + US group, both at 1- and 2-month time points, the newly formed tissues mostly appeared in the background color. Meanwhile, the other group showed highly positive collagen X staining in the newly formed tissue both at 1 or 2 month time points, (Scale bars: 500 μm).

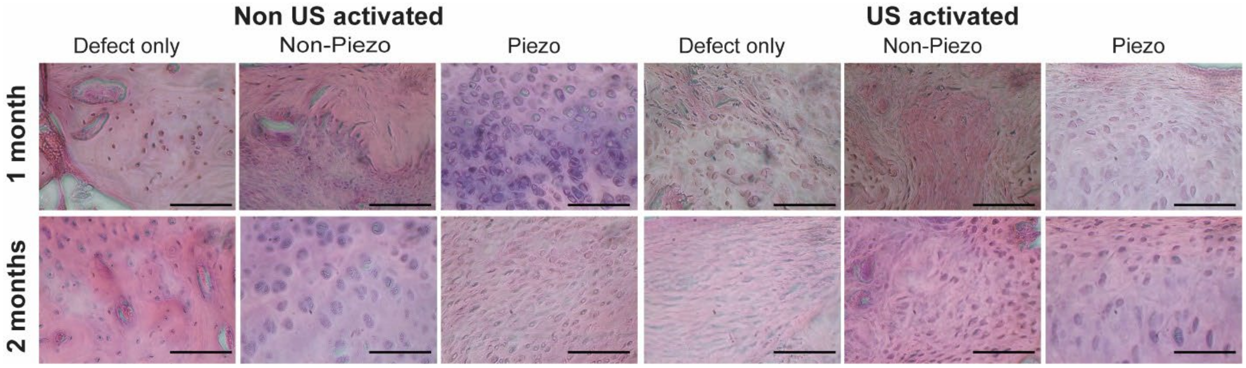

Supplementary Figure 9 | Piezoelectric hydrogel induces cartilage healing evaluated by cell apoptosis using H&E. H&E staining for apoptosis cell visualization at high magnification, for sham (defect only), non-piezo/piezo hydrogels with and without US activation (1-2 months). The apoptotic cells (pyknosis, karyorrhexis) are identifiable as a mass of dark eosinophilic cytoplasm, adopting round or oval shapes, with tightly packed purple or fragmented nuclear chromatin. The pyknosis or karyorrhexis were not observed in any of groups which indicates that hydrogels and US did not induce cells apoptosis (Scale bars: 10 μm).

Supplementary Table 1. ICRS macroscopic evaluation of cartilage repair

Cartilage repair assessment ICRS	Points
Degree of defect repair	
In level with surrounding cartilage	4
75% repair of defect depth	3
50% repair of defect depth	2
25% repair of defect depth	1
0% repair of defect depth	0
Integration to border zone	
Complete integration with surrounding cartilage	4
Demarcating border < 1 mm	3
3/4th of graft integrated, 1/4th with a notable border > 1 mm width	2
1/2 of graft integrated with surrounding cartilage, 1/2 with a notable border > 1 mm	1
From no contact to 1/4th of graft integrated with surrounding cartilage	0
Macroscopic appearance	
Intact smooth surface	4
Fibrillated surface	3
Small, scattered fissures or cracks	2
Several, small or few but large fissures	1
Total degeneration of grafted area	0
Overall repair assessment	
Grade I: normal	12
Grade II: nearly normal	11-8
Grade II: abnormal	7-4
Grade II: severely abnormal	3-1

Supplementary Table 2. ICRS visual histological assessment scale

Feature		Score
I.	Surface	
	Smooth/continuous	3
	Discontinuities/irregularities	0
II.	Matrix	
	Hyaline	3
	Mixture: hyaline/fibrocartilage	2
	Fibrocartilage	1
	Fibrous tissue	0
III.	Cell distribution	
	Columnar	3
	Mixed/columnar clusters	2
	Clusters	1
	Individual cells/disorganized	0
IV.	Cell population viability	
	Predominantly viable	3
	Partially viable	1
	<10% viable	0
V.	Subchondral bone	
	Normal	3
	Increased remodeling	2
	Bone necrosis/granulation tissue	1
	Detached/fracture/callus at base	0
VI.	Cartilage mineralization (calcified cartilage)	
	Normal	3
	Abnormal/inappropriate location	0

Supplementary References

1 ter Haar, G. Therapeutic ultrasound. *European Journal of Ultrasound* **9**, 3-9 (1999). https://doi.org:https://doi.org/10.1016/S0929-8266(99)00013-0

2 Khanna, A., Nelmes, R. T. C., Gougoulias, N., Maffulli, N. & Gray, J. The effects of LIPUS on soft-tissue healing: a review of literature. *British Medical Bulletin* **89**, 169-182 (2008). https://doi.org:10.1093/bmb/ldn040

3 Baek, H., Pahk, K. J. & Kim, H. A review of low-intensity focused ultrasound for neuromodulation. *Biomedical Engineering Letters* **7**, 135-142 (2017). https://doi.org:10.1007/s13534-016-0007-y

4 Xin, Z., Lin, G., Lei, H., Lue, T. F. & Guo, Y. Clinical applications of low-intensity pulsed ultrasound and its potential role in urology. *Transl Androl Urol* **5**, 255-266 (2016). https://doi.org:10.21037/tau.2016.02.04

5 Bikson, M. A review of hazards associated with exposure to low voltages. *New York: University of New York* **20** (2004).

6 Baker, B., Spadaro, J., Marino, A. & Becker, R. O. Electrical stimulation of articular cartilage regeneration. *Ann N Y Acad Sci* **238**, 491-499 (1974). https://doi.org:10.1111/j.1749-6632.1974.tb26815.x

7 Vaca-González, J. J. *et al.* Biophysical Stimuli: A Review of Electrical and Mechanical Stimulation in Hyaline Cartilage. *Cartilage* **10**, 157-172 (2019). https://doi.org:10.1177/1947603517730637

8 Le, T. T. *et al.* Piezoelectric Nanofiber Membrane for Reusable, Stable, and Highly Functional Face Mask Filter with Long-Term Biodegradability. *Advanced Functional Materials* **32**, 2113040 (2022). https://doi.org:https://doi.org/10.1002/adfm.202113040
