# Supplementary material for: Injectable and biodegradable piezoelectric hydrogel for osteoarthritis treatment
Source: Nat Commun. 2023 Oct 6;14:6257. doi: 10.1038/s41467-023-41594-y (PMC10558537; doi:10.1038/s41467-023-41594-y)
Supplement: Supplementary file 2 — Reporting Summary [file 41467_2023_41594_MOESM2_ESM.pdf]

nature portfolio

Corresponding author(s): Thanh D. Nguyen

Last updated by author(s): Sep 5, 2023

Reporting Summary

Nature Portfolio wishes to improve the reproducibility of the work that we publish. This form provides structure for consistency and transparency in reporting. For further information on Nature Portfolio policies, see our Editorial Policies and the Editorial Policy Checklist.

Statistics

For all statistical analyses, confirm that the following items are present in the figure legend, table legend, main text, or Methods section.

n/a	Confirmed	
☐	☒	The exact sample size (n) for each experimental group/condition, given as a discrete number and unit of measurement
☐	☒	A statement on whether measurements were taken from distinct samples or whether the same sample was measured repeatedly
☐	☒	The statistical test(s) used AND whether they are one- or two-sided<br>*Only common tests should be described solely by name; describe more complex techniques in the Methods section.*
☐	☒	A description of all covariates tested
☐	☒	A description of any assumptions or corrections, such as tests of normality and adjustment for multiple comparisons
☐	☒	A full description of the statistical parameters including central tendency (e.g. means) or other basic estimates (e.g. regression coefficient) AND variation (e.g. standard deviation) or associated estimates of uncertainty (e.g. confidence intervals)
☐	☒	For null hypothesis testing, the test statistic (e.g. F, t, r) with confidence intervals, effect sizes, degrees of freedom and P value noted<br>*Give P values as exact values whenever suitable.*
☒	☐	For Bayesian analysis, information on the choice of priors and Markov chain Monte Carlo settings
☒	☐	For hierarchical and complex designs, identification of the appropriate level for tests and full reporting of outcomes
☒	☐	Estimates of effect sizes (e.g. Cohen's d, Pearson's r), indicating how they were calculated

Our web collection on statistics for biologists contains articles on many of the points above.

Software and code

Policy information about availability of computer code

Data collection	PicoScope software (version 6).
Data analysis	Statistics were performed by using GraphPad Prism 9 (v 9.4.1), Dragonfly 2020.1.1.809 (Object Research Systems), MATLAB (2023a), Slicer 5.2.1

For manuscripts utilizing custom algorithms or software that are central to the research but not yet described in published literature, software must be made available to editors and reviewers. We strongly encourage code deposition in a community repository (e.g. GitHub). See the Nature Portfolio guidelines for submitting code & software for further information.

Data

Policy information about availability of data

All manuscripts must include a data availability statement. This statement should provide the following information, where applicable:

- Accession codes, unique identifiers, or web links for publicly available datasets
- A description of any restrictions on data availability
- For clinical datasets or third party data, please ensure that the statement adheres to our policy

All data supporting the findings described in this manuscript are available within the paper, the Supplementary Information, and Source data file. The full image dataset is available from the corresponding author upon request.

Human research participants

Policy information about studies involving human research participants and Sex and Gender in Research.

Reporting on sex and gender	N/A
Population characteristics	N/A
Recruitment	N/A
Ethics oversight	N/A

Note that full information on the approval of the study protocol must also be provided in the manuscript.

Field-specific reporting

Please select the one below that is the best fit for your research. If you are not sure, read the appropriate sections before making your selection.

☒ Life sciences ☐ Behavioural & social sciences ☐ Ecological, evolutionary & environmental sciences

For a reference copy of the document with all sections, see nature.com/documents/nr-reporting-summary-flat.pdf

Life sciences study design

All studies must disclose on these points even when the disclosure is negative.

Sample size	As a standard practice for the field, statistical analysis were performed on data derived from at least 3 biological replicates
Data exclusions	No data were excluded
Replication	Data is derived from at least 3 time repeats, all repeats were successful.
Randomization	Samples were randomly allocated to different experimental groups before treatment. Organisms (i.e., cells) were cultured and maintained in the same environment and randomly allocated to each group
Blinding	For Macroscopic scoring and histological scoring, professionals who evaluated were blinded to all groups. Other than that, blinding was irrelevant to our studies.

Reporting for specific materials, systems and methods

We require information from authors about some types of materials, experimental systems and methods used in many studies. Here, indicate whether each material, system or method listed is relevant to your study. If you are not sure if a list item applies to your research, read the appropriate section before selecting a response.

Materials & experimental systems

n/a	Involved in the study	
☐	☒	Antibodies
☐	☒	Eukaryotic cell lines
☒	☐	Palaeontology and archaeology
☐	☒	Animals and other organisms
☒	☐	Clinical data
☒	☐	Dual use research of concern

Methods

n/a	Involved in the study	
☒	☐	ChIP-seq
☒	☐	Flow cytometry
☒	☐	MRI-based neuroimaging

Antibodies

Antibodies used	AF5710 (1:100, OriGene)<br>Alexa Fluor 488 labeled goat anti-mouse IgG (H+L) secondary antibody (1:2000) Invitrogen.<br>ab49945 (1:200) and ab34712 (1:100) from Abcam
Validation	Antibodies were validated by the manufacturer or other publications.<br>References and manufacturer validations can be found as below:

AF5710 - https://m1.acris-antibodies.com/pdf/AF5710.pdf
Alexa Fluor 488 labeled goat anti-mouse IgG (H+L) secondary antibody- https://www.thermofisher.com/antibody/product/Goat-anti-Mouse-IgG-H-L-Cross-Adsorbed-Secondary-Antibody-Polyclonal/A-11001.
ab49945- https://www.abcam.com/products/primary-antibodies/collagen-x-antibody-col-10-ab49945.html
ab34712 - https://www.abcam.com/products/primary-antibodies/collagen-ii-antibody-ab34712.html

Eukaryotic cell lines

Policy information about cell lines and Sex and Gender in Research

Cell line source(s)	Human THP-1 was obtained from American Type Culture Collection (ATCC) from 1 year old male patient.<br>Rabbit Adipose-derived stem cells (ADSCs) RBXMD-01001 was obtained from Cyagen
Authentication	Cells were used without modification after receiving from the supplier and therefore were not authenticated
Mycoplasma contamination	The cell line was tested monthly to be negative for mycoplasma contamination.
Commonly misidentified lines (See ICLAC register)	There was no commonly misidentified cell lines that used in our research.

Animals and other research organisms

Policy information about studies involving animals; ARRIVE guidelines recommended for reporting animal research, and Sex and Gender in Research

Laboratory animals	New Zealand white rabbits weighing ~ 3 kg were obtained Envigo
Wild animals	The study did not use wild animals.
Reporting on sex	The finding applies only on male animals.
Field-collected samples	The study did not involve samples collected from the field.
Ethics oversight	All the animal studies were approved by the University of Connecticut Health Institutional Animal Care and Use Committee (IACUC)-protocol #TE-102090-0622 and AP-200653

Note that full information on the approval of the study protocol must also be provided in the manuscript.
