# Supplementary material for: Injectable and biodegradable piezoelectric hydrogel for osteoarthritis treatment
Source: Nat Commun. 2023 Oct 6;14:6257. doi: 10.1038/s41467-023-41594-y (PMC10558537; doi:10.1038/s41467-023-41594-y)
Supplement: Supplementary file 3 — Description of Additional Supplementary Files [file 41467_2023_41594_MOESM3_ESM.pdf]

Title: Supplementary Movie 1:
Description: Injecting Piezoelectric hydrogel with X RAY guidance.

Title: Supplementary Movie 2:
Description: Injection NFs-PLLA hydrogel using a G-29 needle to show injection ability of the hydrogel with G29 needle.

Title: Supplementary Movie 3:
Description: Rabbit under US activation treatment.

Title: Supplementary Movie 4:
Description: Rabbit behaves normally after US activation treatment.
